# Small bowel herniation through the epiploic foramen reduced laparoscopically

**DOI:** 10.1308/rcsann.2025.0053

**Published:** 2025-07-15

**Authors:** S Bhattacharyya, M Bassuni

**Affiliations:** ^1^ Bedfordshire Hospitals NHS Foundation Trust, UK; ^2^ Cambridge University Hospitals NHS Foundation Trust, UK

**Keywords:** Internal hernia, Laparoscopy, Intestinal obstruction, Peritoneal cavity

## Abstract

Herniation of intestinal contents through the epiploic foramen is a rare phenomenon. Diagnosis is often missed owing to its non-specific presentation, delaying management and increasing morbidity and mortality. We report a 60-year-old man presenting with symptoms of abdominal pain and total constipation with small bowel dilatation and unclear computed tomography findings. Exploratory laparoscopy revealed terminal ileum herniation through the epiploic foramen. Laparoscopic reduction was performed with no bowel resection required. Postoperative recovery was uneventful with no recurrence. Controversy exists in the literature regarding closure of the foramen to prevent recurrence; however, this carries significant risks. Repeated assessment and early exploration are key in identifying and managing such rare presentations.

## Background

The epiploic foramen is a natural opening allowing communication between the peritoneal cavity and lesser sac. Anatomically, the caudate lobe lies superiorly, duodenum inferiorly, hepatoduodenal ligament anteriorly and inferior vena cava posteriorly.^1^ There is potential for intestinal contents to herniate through the foramen, although this is incredibly rare, accounting for 0.2%–0.9% of all hernias.^2,3^ This herniation can be classified into four types (Table 1) with the small bowel being the most common structure to herniate.^3^ Presentation is often non-specific, ranging from diffuse abdominal pain to signs of intestinal obstruction.^4^ Abdominal computed tomography (CT) scans with contrast are the modality of choice for diagnosis; however, radiological signs can be subtle or unclear.^3,5^ In addition, owing to the narrow anatomy of the foramen, there is a high chance of bowel becoming strangulated.^6^ Non-specific presentations with unclear radiology can delay diagnosis and management. These factors lead to a higher chance of bowel ischaemia and perforation, increasing the hernia's associated morbidity and mortality rates.^5^ There is a need for awareness of the hernia itself, analysis of subtle findings on imaging, consideration of developmental risk factors and prevention of future recurrence, with the latter being a controversial topic in the literature.

**Table 1:** Types of epiploic hernias and the percentage of each reported in the literature^3^

Type of herniation through the epiploic foramen	Contents	Percentage of cases
1	Small bowel only	63–65
2	Terminal ileum, caecum and ascending colon	25–30
3	Transverse colon	7
4	Gallbladder or other intraperitoneal structures	1–3

## Case history

**Figure 1:**
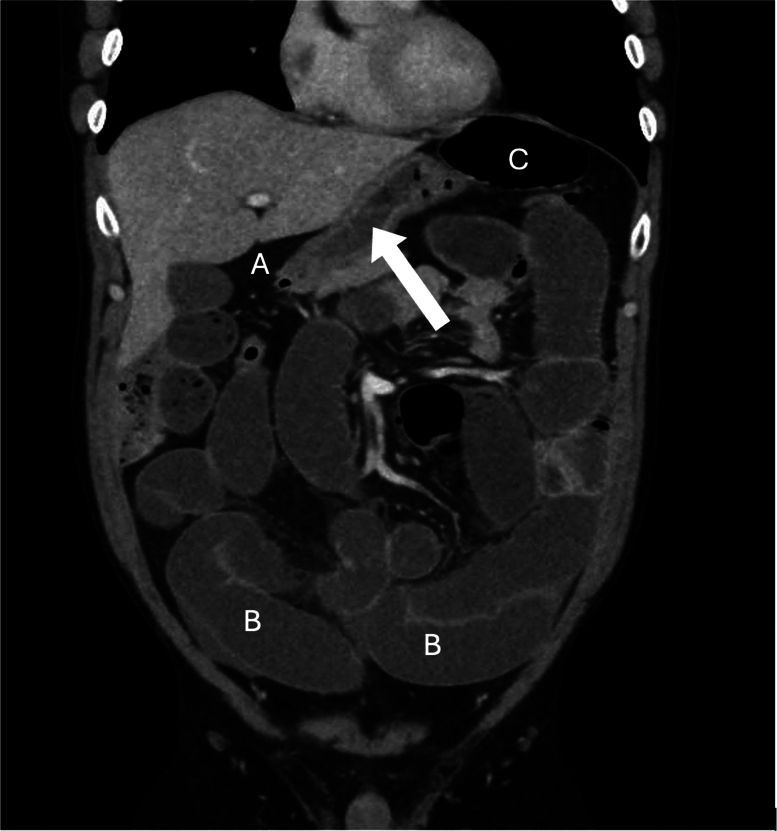
Coronal section of a computed tomography scan showing terminal ileum herniation through the foramen of Winslow (A) with displacement of the stomach (B) and dilated small bowel loops (C). The white arrow denotes terminal ileum herniation through the foramen of Winslow.

**Figure 2:**
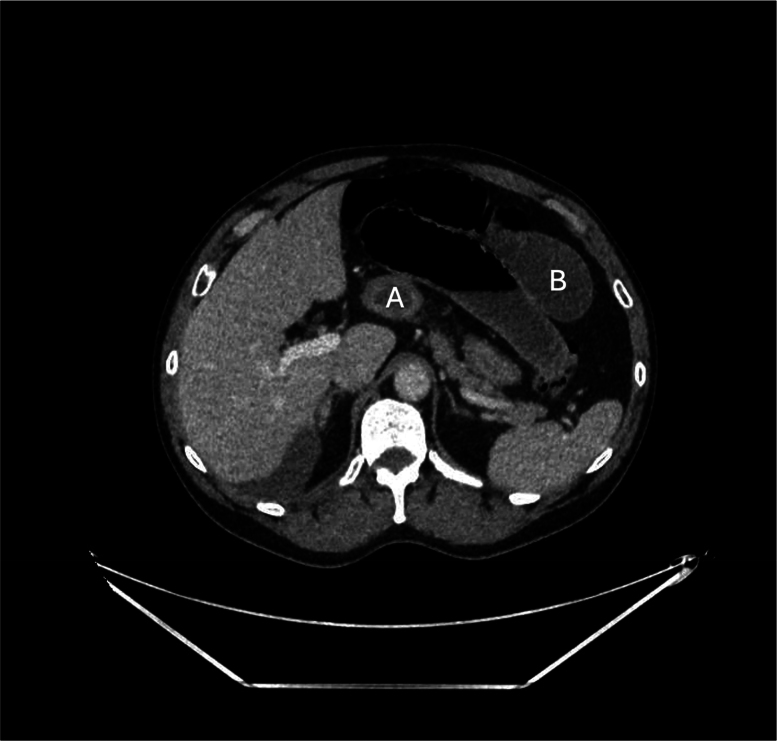
Axial section of computed tomography scan showing terminal ileum (A) herniation through the epiploic foramen (foramen of Winslow), with dilated small bowel loops (B).

We report a 60-year-old man presenting with a 1-day history of generalised abdominal pain with distention, absolute constipation and persistent vomiting. His vital signs were stable and he had no past surgical history. Blood sciences revealed raised inflammatory markers with a white cell count of 13.1×10^9^/L and C-reactive protein of 26mg/L. Abdominal CT scan with contrast showed severe small bowel dilation with a transition point at the terminal ileum, which also appeared tethered towards the sub-hepatic region, abnormal caecal pole position and displacement of the stomach superiorly (Figures 1 and 2). Early vascular compromise was noted by the radiologist. Initial management was initiated with intravenous fluids, analgesia, anti-emetics, antibiotics and nasogastric (NG) tube insertion. Reassessment of the patient the following morning revealed a further distended, rigid abdomen with absent bowel sounds and continued absolute constipation. The NG tube aspirated only 100ml and despite this, the patient reported continuous vomiting episodes with the tube in situ. A venous blood gas revealed a blood lactate level of 1.9mmol/L. Owing to the patient's worsening condition and an unclear source for the obstruction on CT, a decision was made to undertake an emergency diagnostic laparoscopy the same day.

Direct visualisation on laparoscopy revealed the terminal ileum herniating through the epiploic foramen to be the cause of obstruction. Laparoscopic reduction was performed using gentle traction. There was no abnormal widening of the epiploic foramen, hypermobile bowel or evidence of malrotation present on inspection. The bowel was deemed to be fully viable after being checked over 10min with no resection required. We did not repair the epiploic foramen closed. The patient had an uneventful postoperative period with a marked improvement in symptoms and was discharged on day 2 postoperatively. On 6-month follow-up, the patient has not reported any further symptoms or recurrence.

## Discussion

The epiploic hernia is a rare but important presentation posing certain challenges for surgeons. Patients often present with non-specific symptoms, making diagnosis difficult based on symptoms alone. Some reports mention epigastric or right upper quadrant pain, whereas in our case and others, patients may have generalised pain associated with distension, absolute constipation and persistent vomiting.^1–^^8^ Colicky pain may arise secondary to a hernia that intermittently obstructs and self-reduces.^3^ Most cases do not have a significant surgical history, eliminating adhesive bowel obstruction from the list of differentials. Imaging is often performed as an adjunct for diagnostic clues.^2,6,8^

Abdominal CT scan with contrast is the modality of choice for preoperative diagnosis of the epiploic hernia. Certain clues can be elicited from CT imaging, including dilated small bowel loops extending towards the liver and inferior vena cava.^1–4,6–8^ A distended stomach displaced anteriorly and laterally may be identified, similar to our findings (Figures 1 and 2).^3,5^ Stretching or swirling of mesenteric vessels may indicate vascular compromise, a potential indicator for impending bowel ischaemia.^8^ Many times, it is not possible to identify an obvious cause for obstruction on CT, warranting urgent surgical exploration.^1–3^

There has been a shift from laparotomy to laparoscopy for the diagnosis and treatment of internal epiploic hernias. In our case, laparoscopy alone was utilised with gentle traction for reduction with no bowel resection required. There have been two similar cases to ours but both other older and newer reports have often utilised laparotomy and required bowel resection.^1,2,4–8^ If reduction is difficult, needle decompression or the Kocher manoeuvre can be utilised, the latter involving division of the gastrocolic ligament to widen the epiploic foramen to release herniated bowel.^3^ We hypothesise that resection was avoided because of prompt imaging in conjunction with clinical assessment that identified the deteriorating patient, allowing explorative laparoscopy on day 2 of admission. In addition, our patient did not have specific risk factors for development of the hernia itself, potentially avoiding a difficult reduction experience.

Several risk factors for the development of epiploic hernias have been hypothesised. If the foramen is enlarged (defined genetically), around 3cm or more, this is enough for viscera to herniate through and appears to be the strongest risk factor for epiploic herniation.^3,6^ Other risk factors include hypermobile viscera due to long mesentery, intra-abdominal pressure changes, mobility of the caecum and malrotation.^6^ Our patient, however, had none of the risk factors above, particularly with a foramen less than 3cm (1–2 finger breadths wide). This led to us not closing the foramen, a decision that is controversial in the literature.

There is no consensus regarding foramen closure in preventing the risk of recurrence for epiploic hernias. There have been no recent reports of recurrence irrespective of foramen closure.^1–3,7^ Closure of the foramen is associated with damage to surrounding structures including the portal vein, hepatic artery and bile duct.^8^ It has been argued inflammatory and adhesive processes will close the gap in the postoperative period, eliminating the need for closure.^4^ Despite the risks, some surgeons have opted to close the foramen, particularly if an enlarged foramen was present.^2^ Because none of the above risk factors were present, we opted to leave the foramen open. At 6-month follow-up, our patient was healthy with no evidence of recurrence.

## Conclusion

The epiploic hernia is rare, presents non-specifically and carries a high risk of strangulation. CT scans may provide clues to allow for preoperative diagnosis but laparoscopy is likely to provide a definitive diagnosis. Various reduction techniques have been reported including the Kocher technique and gentle reduction, with or without bowel resection. Intraoperatively, it is important to assess for any risk factors such as enlarged foramen, mesentery or rotation that could have led to the internal hernia. Recurrence of the epiploic hernia remains a controversy; however, our and other recent cases suggest recurrence is unlikely regardless of whether the foramen is closed or not. Key learning points from this report include the importance of regular reassessment of the deteriorating patient, early CT imaging to identify symptom source and awareness of the epiploic hernia itself to aid future surgeons in diagnosis intraoperatively and subsequent management to prevent associated morbidity.
